# The prolyl hydroxylase enzymes are positively associated with hypoxia-inducible factor-1α and vascular endothelial growth factor in human breast cancer and alter in response to primary systemic treatment with epirubicin and tamoxifen

**DOI:** 10.1186/bcr2825

**Published:** 2011-02-03

**Authors:** Stephen B Fox, Daniele Generali, Alfredo Berruti, Maria P Brizzi, Leticia Campo, Simone Bonardi, Alessandra Bersiga, Giovanni Allevi, Manuela Milani, Sergio Aguggini, Teresa Mele, Luigi Dogliotti, Alberto Bottini, Adrian L Harris

**Affiliations:** 1 Peter MacCallum Cancer Centre, St Andrews Place, East Melbourne, Victoria 3002, Australia; 2 Unità di Patologia Mammaria - Breast Cancer Unit and Anatomia Patologica, Azienda Instituti Ospitalieri di Cremona, Viale Concordia 1, 26100 Cremona, Italy; 3 Oncologia Medica, Dipartimento di Scienze Cliniche e Biologiche, Università di Torino Azienda Ospedaliera San Luigi di Orbassano, Regione Gonzole 10, Orbassano 10043, Italy; 4 Molecular Oncology Laboratories, Weatherall Institute of Molecular Medicine, University of Oxford, John Radcliffe Hospital, Oxford OX3 9DS, UK

## Abstract

**Introduction:**

The purpose of the present study was to investigate the relationship of expression of hypoxia inducible factor (HIF)-1α-modifying enzymes prolyl hydroxylase (PHD)1, PHD2 and PHD3 to response of tumours and survival in breast cancer patients enrolled in a phase II trial of neoadjuvant anthracycline and tamoxifen therapy.

**Methods:**

The expression of PHD1, PHD2 and PHD3 together with HIF-1α and the HIF-inducible genes vascular endothelial cell growth factor (VEGF) and carbonic anhydrase IX were assessed by immunohistochemistry using a tissue microarray approach in 211 patients with T2-4 N0-1 breast cancer enrolled in a randomised trial comparing single-agent epirubicin versus epirubicin and tamoxifen as the primary systemic treatment.

**Results:**

PHD1, PHD2 and PHD3 were detected in 47/179 (26.7%), 85/163 (52.2%) and 69/177 (39%) of tumours at baseline. PHD2 and PHD3 expression was moderate/strong whereas PHD1 expression was generally weak. There was a significant positive correlation between HIF-1α and PHD1 (*P *= 0.002) and PHD3 (*P *< 0.05) but not PHD2 (*P *= 0.41). There was a significant positive relationship between VEGF and PHD1 (*P *< 0.008) and PHD3 (*P *= 0.001) but not PHD2 (*P *= 0.09). PHD1, PHD2 and PHD3 expression was significantly increased after epirubicin therapy (all *P *< 0.000) with no significant difference in PHD changes between the treatment arms. There was no significant difference in response in tumours that expressed PHDs and PHD expression was not associated with survival.

**Conclusions:**

Although expression of the PHDs was not related to response or survival in patients receiving neoadjuvant epirubicin, our data provide the first evidence that these enzymes are upregulated on therapy in breast cancer and that the biological effects independent of HIF make them therapeutic targets.

## Introduction

The hypoxic response through hypoxia inducible factor (HIF) is recognised as one of the most important microenvironmental influences on tumour behaviour that enables tumours to acquire an aggressive phenotype and become resistant to both chemotherapy and radiotherapy [1,2]. Although HIF-1α is transcribed continuously, in normoxia its levels are kept low by a rapid degradation process through the ubiquitin-proteasome system [3]. This process is achieved through hydroxylation of two prolyl residues at Pro-402 and Pro-564 in the oxygen-dependent HIF-1α [4], and leads to recognition by the von Hippel-Lindau tumour suppressor protein and targeting for degradation [5]. Three isoenzymes - prolyl hydroxylase (PHD)1, PHD2 and PHD3 - are responsible for the modification of HIF-1α, the activity of which is dependent on the presence of oxygen as a co-substrate together with iron, ascorbate and 2-oxoglutarate as essential co-factors [6]. Therefore as the abundance of molecular oxygen for hydroxylation decreases, which occurs in the hypoxic microenvironment of a tumour with its disordered and leaky vasculature, a reduction in PHD enzymatic activity occurs, allowing HIF-1α accumulation. HIF-1α is then able to translocate to the nucleus, where it dimerises with its constitutively expressed partner HIF-1β (also known as aryl nuclear hydrocarbon translocator) and then binds to the hypoxic response element of genes that enhance tumour cell survival such as glycolysis (Glut1), angiogenesis (for example, vascular endothelial growth factor (VEGF)), iron metabolism (transferrin), pH control (carbonic anhydrase (CAIX)) and haemoglobin synthesis (erythropoietin) (reviewed in [1]).

HIF-1α and its downstream genes such as VEGF and CAIX are associated with advanced tumour stage, metastases and a shorter survival in breast cancer [7-9]. The HIF pathway also upregulates several genes that lead to a proapoptotic phenotype, showing the complicated effects of HIF in tumours. This paradox is observed in breast cancer, where there appears to be a pivotal switch in the HIF-induced gene for BNIP3 with progression from *in situ *to invasive carcinoma [10]. This apparent dual action of HIF is also emphasised in tumour models that have demonstrated HIF is able to enhance both tumour growth and angiogenesis, and also in data to show that HIF has tumour-suppressive activity [11-13]. Since the PHDs directly regulate and are regulated by HIF-1α, and have been shown to control a number of other significant intracellular factors including the adrenergic receptor and NF-κB [14], the above apparent opposing actions potentially being due to the action of the PHDs.

Although many of the downstream HIF genes have been studied extensively in breast tumours, however, there are limited data for the PHDs [15]. We therefore decided to assess the role of the PHDs in breast cancer patients treated by neoadjuvant chemotherapy in the setting of a phase II randomised trial. This assessment provides the optimal opportunity for investigating the effect of the PHDs, since HIF has been shown to mediate resistance to chemotherapy and radiotherapy and biopsies can be compared both pre treatment and post treatment. Our aims were to investigate the expression of the regulatory hydroxylases, PHD1, PHD2 and PHD3 in tumours from patients at baseline and post treatment, to correlate the expression of these factors with clinicopathological parameters, to explore the potential of these factors as biomarkers for predicting tumour response, and to explore the association between changes in their expression and survival.

## Materials and methods

### Patients

Patients with T2-4 N0-1 breast cancer were recruited into a randomised trial comparing single-agent epirubicin (EPI arm) versus epirubicin plus tamoxifen (EPI-TAM arm) as the primary systemic treatment [16]. Patients were accrued from January 1997 to December 2001. The study was approved by the Institutional Ethics Committee. All patients gave written informed consent to the diagnostic procedures, the proposed treatment, and the biological evaluations. Two hundred and eleven patients were enrolled: 105 were randomised to receive epirubicin alone, and 106 were randomised to receive epirubicin plus tamoxifen.

On first presentation, an incision biopsy was performed on each patient and a small tissue sample (5 to 8 mm) was removed. Chemotherapy was started within 2 days of diagnosis. Patients in the EPI arm received 60 mg/m^2 ^epirubicin (Farmorubicina; Pharmacia, Milan, Italy) by slow intravenous push on days 1 and 2; whereas patients in the EPI-TAM arm received 60 mg/m^2 ^epirubicin by slow intravenous push on days 1 and 2 and 30 mg tamoxifen (Kessar; Pharmacia) daily. Epirubicin injections were repeated every 21 days for three or four cycles before definitive surgery, whereas tamoxifen was given continuously until definitive surgery. All patients postoperatively received four cycles of the CMF regimen: intravenous cyclophosphamide 600 mg/m^2^, intravenous methotrexate 40 mg/m^2^, and intravenous 5-fluorouracil 600 mg/m^2 ^on days 1 and 8, every 28 days [11]. Patients with oestrogen receptor (ER)-positive primary tumour in both treatment arms received tamoxifen (20 mg; that is, lower than the primary dose) starting after surgery, up to progression or for a maximum of 5 years. The median follow-up of patients was 53 months (August 2004; range, 13 to 95 months).

For treatment evaluation, the same clinician used a calliper measuring the size of the primary tumour monthly and manually the size of the axillary lymph nodes, when appreciable. Response was assessed before definitive surgery by clinical measurement of the changes in the product of the two largest diameters recorded in two successive evaluations. According to World Health Organisation criteria, tumour progression was defined as an increase of at least 25% in tumour size; stable disease was defined as an increase <25% or a reduction <50%; partial response was defined as tumour shrinkage >50%; and complete response was defined as the complete disappearance of all clinical signs of disease [17]. Pathologic complete response was defined as the absence of neoplastic cells in the breast and in the axillary lymph nodes. Surgery was planned after full clinical reassessment. Quadrantectomy or modified radical mastectomy was carried out when indicated in association with full axillary node dissection. All patients subjected to quadrantectomy underwent irradiation of the residual breast (60 Gy delivered over 6 weeks).

### Histopathologic grade and immunohistochemistry

Tumour grade was evaluated using the modified Bloom and Richardson method. Immunohistochemical evaluation was performed on paraffin-embedded tumour samples obtained at diagnosis and at definitive surgery. Bcl2, p53, ER, progesterone receptor, and Ki67 staining were carried out at the Pathology Unit of the Azienda Ospedaliera Istituti Ospitalieri of Cremona (Italy). The immunohistochemical method used for routine markers is fully described elsewhere [18].

Immunohistochemistry for PHD1, PHD2, and PHD3 was performed on sections from tissue microarrays containing two 1 mm tumour cores taken from selected morphologically representative tumour regions of each paraffin-embedded breast tumour from both the initial diagnostic incisional biopsy and from tumour remaining at definitive surgery. Quality control was assessed on each block by haematoxylin and eosin staining. The tissue microarray sections were cut from each block at 4 μm thick intervals, dewaxed, placed through graded alcohol and placed into water. Antigen retrieval was performed in PT Link (Dako, Glostrup, Denmark) using EnVision FLEX Target Retrieval Solution (Dako) - low pH for PHD1, PHD2 and PHD3, and high pH for HIF-1α - for 20 minutes at 100°C. VEGF required antigen retrieval in pH 8 buffer (20 mM Tris/1 mM ethylenediamine tetraacetic acid/10 mM sodium citrate) for 2 minutes in a pressure cooker. Endogenous peroxidase was blocked with EnVision FLEX Peroxidase-Blocking Reagent (Dako) before incubating the sections with respective monoclonal antibodies, which were produced by our group. The antibodies used to detect the hydroxylases were PHD1 (112), PHD2 (366G/76) and PHD3 (EG188e), which have previously been validated [15]. Slides were counterstained with haematoxylin and were mounted.

### Immunohistochemical scoring criteria

Staining for bcl2, p53 and Ki67 was scored by counting the number of positively stained cells and was expressed as a percentage of the total tumour cells (at least 1,000) counted across several representative fields of the section using a standard light microscope equipped with a 10 × 10 square graticule. Reproducibility of counting was assessed by a second investigator rescoring 10 slides [19,20]. Cut-off values of the median values in each group were compared. ER and progesterone receptor were assessed in a semiquantitative fashion as described previously by McCarty and colleagues [21], incorporating both the intensity and distribution of specific staining. The H-score was derived from the sum of the percentages of positive-stained epithelial cells multiplied by the weighted intensity of staining. Specimens were deemed receptor positive if the H-score was greater than 100. The immunohistochemical evaluation at mastectomy was performed by the same pathologists, who remained blinded as regards the disease response and the score assessed at first biopsy.

Scoring for PHDs and VEGF was carried out according to the previously used semiquantitative system [15,22,23]. Although nuclear staining for the PHDs occurred, this was at low frequency; scoring of the percentage of cells expressing the protein and of the intensity of staining in the cytoplasm was therefore performed for PHDs and VEGF. The scoring system for intensity was: 0 = no staining, 1 = weak staining, 2 = moderate staining, 3 = strong staining. The scoring system for percentage was: 0 = no cells staining positive, 1 = <10% cells staining positive, 2 = 11 to 50% positive cells, 3 = 51 to 80% positive cells, 4 = >80% positive cells. At least 10% of cells needed to be stained to be considered positive.

HIF-1α was scored only according to the presence (1+) or absence (0) of nuclear expression. For CAIX membrane staining, a score of 0 to 3 for the intensity was given (0 = no staining, 1 = weak staining, 2 = moderate staining, 3 = strong staining). For HIF and CAIX any staining was considered positive, as hypoxia is frequently focal. The missing cases indicated in Table 1 were not scored due to an absent core or an insufficient number of tumour cells present in the tissue microarray core (see Additional file 1).

**Table 1:** Distribution of immunoscoring for hypoxia pathway factors and cut-off values used in semiquantitative analyses

Factor	*n *(%)
Hypoxia-inducible factor-1α	
0	33 (19.3%)
1	100 (58.5%)
2	38 (22.2%)
Missing	16
Vascular endothelial growth factor	
0	32 (20.0%)
1	39 (24.4%)
2	37 (23.1%)
4	52 (32.5%)
Missing	27
Carbonic anhydrase IX	
0	125 (75.3%)
1	20 (12.1%)
≥2	21 (12.6%)
Missing	21
Prolyl hydroxylase 1	
0	129 (73.3%)
1	43 (24.4%)
2	3 (1.7%)
3	1 (0.6%)
Missing	11
Prolyl hydroxylase 2	
0	78 (47.8%)
1	51 (31.3%)
2	30 (18.4%)
3	4 (2.4%)
Missing	24
Prolyl hydroxylase 3	
0	108 (61.0%)
1	53 (29.9%)
2	15 (8.5%)
3	1 (0.6%)
Missing	10

Data refer to the number of tumours positive in each intensity score category.

### Statistical methods

The chi-square test, the chi-square test for trend, and Fisher's Exact test were used when indicated to perform comparisons of proportions. Kruskal-Wallis analysis of variance was performed to compare continuous variables. Correlations between discrete variables were made by Spearman's *R *test for nonparametric data. Comparison of discrete data in matched tumour samples was done using the Wilcoxon rank-sum test for paired data. Overall survival was calculated from randomisation to the occurrence of disease relapse or disease-related death. Patients were censored if they were free from recurrence and alive at the last follow-up period. Overall survival curves were estimated using the Kaplan-Meier method. Unadjusted differences in these estimates were assessed with the log-rank test. All *P *values reported were two-sided; *P *< 0.05 was considered statistically significant. The Statistica for Windows software package (version 8, StatSoft, Tulsa, OK, USA) was used for statistical analyses.

## Results

### Patient characteristics

One hundred and eighty-seven of the 211 (88.6%) patients prospectively enrolled in the trial were evaluable for PHD1, PHD2 and PHD3 staining. For the remaining 24 patients, insufficient material was available for the study. Patient characteristics are shown in Table S1 in Additional file 1. Ninety patients (48%) were randomised to the EPI arm and 97 (52%) patients were randomised to the EPI-TAM arm. PHD1 was evaluated at baseline excision in 176 (94%) tumours, PHD2 in 163 (87%) tumours and PHD3 in 177 (94%) tumours. The corresponding numbers of marker evaluation in matched tumour samples at biopsy and main tumour resection were 130 (69%) tumours, 111 (59%) tumours and 127 (67.9%) tumours, respectively (Figure S1 in Additional file 1). There were 12 patients who had a complete pathological response.

### Relationship between tumours expressing PHD1, PHD2 and PHD3 at baseline and clinicopathologic variables

PHD1 was expressed in 47/176 (26.7%) tumours, PHD2 in 85/163 (52.2%) tumours and PHD3 in 69/177 (39%) tumours at baseline. The distribution of expression differed for the different PHDs such that most positivity for PHD1 was weak whereas for PHD2 and PHD3 the dynamic range of expression was wider, being frequently moderate to strong. Since the dynamic range (the range of expression observed in this series) of the PHD1 was low, PHD1 stratified as only negative (no staining) and positive (>0 staining) whereas PHD2 and PHD3 were stratified semiquantitatively (0 to 3). There was an inverse relationship between both PHD1 and PHD3 positivity and high tumour grade (*P *< 0.03 and *P *= 0.04, respectively), but no significant relationship was observed between PHD1 or PHD2 expression and HER2, T status, N status, p53, bcl2, Ki67, ER or progesterone receptor (*P *> 0.05; see Tables S2 to S4 in Additional file 1). PHD3 expression did not show any relationship with any of the clinical and biological parameters considered. No association was observed between tumours that express all PHDs and tumour size, grade, nodal status, ER, progesterone receptor, HER2, p53, ki67, bcl2 or ki67 (all *P *> 0.05; see Table S5 in Additional file 1).

### Relationship of tumours expressing PHD1, PHD2 and PHD3 at baseline with HIF-1α and HIF-induced markers

There was a significant positive relationship between HIF-1α and PHD1 (*P *= 0.002) and PHD3 (*P *< 0.05) but not PHD2 (*P *= 0.41). There was a significant positive relationship between VEGF and PHD1 (*P *< 0.008) and PHD3 (*P *= 0.001) but not PHD2 (*P *= 0.09). There was no significant association between CAIX and PHD1, PHD2 or PHD3 (all *P *> 0.05).

### Effect of treatment on tumours expressing PHD1, PHD2 and PHD3

PHD1, PHD2 and PHD3 expression was significantly increased after therapy with epirubicin either alone or in combination with tamoxifen (*P *< 0.0001, *P *< 0.0001 and *P *< 0.0001) (Table 2). PHD1 positivity was thus present in 43/130 baseline tumour samples (33.1%) but in 111/130 tumour samples (85.4%) at residual tumour histology. Similar results were obtained for PHD2, where 49/111 (44.1%) tumour samples were positive at baseline and 98/111 (88.3%) tumour samples were positive after chemotherapy. PHD3 was positive in 58/127 (45.6%) tumour samples at baseline and in 119/127 (93.7%) tumour samples after chemotherapy. There was no significant difference in PHD changes between the treatment arms, or between tumours stratified according to the ER status and treatment administered in ER-positive patients (all *P *> 0.05).

**Table 2:** PHD1, PHD2 and PHD3 expression before and after treatment in matched cases

		Before *n *(%)	After *n *(%)	*P *value
PHD1	Overall (*n *= 130)			
	0	87 (66.9)	19 (14.6)	0.0000
	1	39 (30.0)	68 (53.3)	
	2	3 (2.3)	36 (27.7)	
	3	1 (0.8)	7 (5.4)	
	EPI (*n *= 64)			
	0	40 (62.5)	8 (12.5)	0.0000
	1	23 (35.9)	32 (50.0)	
	2	1 (1.6)	23 (35.9)	
	3		1 (1.6)	
	EPI-TAM (*n *= 66)			
	0	47 (71.2)	11 (16.7)	0.0000
	1	16 (24.2)	36 (54.5)	
	2	2 (3.0)	13 (19.7)	
	3	1 (1.5)	6 (9.1)	
PHD2	Overall (*n *= 111)			
	0	62 (55.9%)	13 (11.7%)	0.0000
	1	31 (27.9%)	15 (13.6%)	
	2	16 (14.4%)	43 (38.7%)	
	3	2 (1.8%)	40 (36.0%)	
	EPI (*n *= 59)			
	0	31 (52.5)	5 (8.5)	0.0000
	1	16 (27.1)	6 (10.2)	
	2	10 (16.9)	22 (37.3)	
	3	2 (3.4)	26 (44.1)	
	EPI-TAM (*n *= 52)			
	0	31 (59.6)	8 (15.4)	0.0000
	1	15 (28.8)	9 (17.3)	
	2	6 (11.5)	21(40.4)	
	3	0	14 (26.9)	
PHD3	Overall (*n *= 127)			
	0	69/127 (54.3%)	8/127 (6.3%)	0.00
	1	46/127 (36.2%)	47/127 (37.0%)	
	2	12/127 (9.4%)	47/127 (37.0%)	
	3	0.00	25/127 (19.7%)	
	EPI (*n *= 63)			
	0	29/63(46.0%)	3/63(4.8%)	0.00
	1	27/63 (42.8%)	22/63 (34.9%)	
	2	7/63 (11.1%)	23/63 (36.5%)	
	3	0.00	15/63 (23.8%)	
	EPI-TAM (*n *= 64)			
	0	40/64 (62.5%)	5/64 (7.8%)	0.00
	1	19/64 (29.7%)	25/64 (39.1%)	
	2	5/64 (7.8%)	24/64 (37.5%)	
	3	0.00	10/64 (15.6%)	

EPI, epirubicin treatment; EPI-TAM, epirubicin plus tamoxifen treatment; PHD, prolyl hydroxylase.

### Predictive role of PHD1, PHD2 and PHD3 expression and treatment activity

The relationship between baseline PHD1, PHD2 and PHD3 status and treatment response is depicted in Table 3. PHD1 and PHD3 positivity showed a progressive decrease according to the grade of response obtained, but this failed to attain statistical significance (*P *= 0.15 and *P *= 0.14, respectively). PHD2 positivity showed a similar but increasing nonsignificant trend with tumour response (*P *= 0.17). There was no significant difference in response in tumours that expressed all PHDs (*P *= 0.59).

**Table 3:** Predictive role of baseline PHD1, PHD2 and PHD3 positivity for disease response in overall cases

	No response	Partial Response	Complete Response	*P *value
PHD1-positive	13/38 (34.2%)	28/105 (26.7%)	6/32 (18.7%)	0.15
PHD2-positive	18/35 (51.4%)	45/97 (46.4%)	21/30 (70.0%)	0.17
PHD3-positive	19/38 (50.0%)	39/105 (37.1%)	11/33 (33.3%)	0.14

One patient was not assessable for disease response due to treatment refusal after one cycle.

### Prognostic role of PHD1, PHD2 and PHD3

There was no significant difference in disease-free survival at baseline histology or residual histology for patients with tumours expressing PHD1 (*P *= 0.17 and *P *= 0.23, respectively), PHD2 (*P *= 0.91 and *P *= 0.11, respectively) or PHD3 (*P *= 0.42 and *P *= 0.12, respectively). There was no significant difference in disease-free survival when stratifying patients by their tumours expressing all PHDs either at baseline (*P *= 0.76) or on residual histology (*P *= 0.22).

## Discussion

HIF signalling is critically important for cell survival in low-oxygen environments, as occurs in tumours [24]. HIF is rapidly degraded after hydroxylation by the PHDs in the presence of molecular oxygen [25]. We hypothesised that tumours with high baseline levels of the PHDs and/or that are able to induce PHD after chemotherapy could modulate HIF-1α levels and thereby alter HIF signalling. The resultant effect could change the biological behaviour of the tumour and may be useful as a predictive marker. We also hypothesised that certain chemotherapeutic agents may be able to alter PHDs expression in tumours either directly through their effect on tumour cells or indirectly through changes in vascularity and oxygen delivery, and thereby also modulate HIF activity.

We observed frequent expression of the PHDs in invasive breast carcinoma that ranged from ~25 to 50% of cancer cells, suggesting that the PHDs are important in human breast cancer. Expression of PHD2 and PHD3 was stronger than PHD1, which is in keeping with these being the most potent isoforms that regulate HIF-1α and HIF-2α [26] - although PHD1 (and PHD3) may cooperate with PHD2, the major oxygen sensor [27]. Although PHD1 levels are regulated by oestrogen [28], we observed no association between ER and PHD1 either at baseline or in patients treated with tamoxifen - suggesting this is not a major control mechanism for PHD1 in breast cancer. This observation also supports the notion of PHD1 not having a significant role in breast tumorigenesis. Nevertheless, PHD1 and PHD3 were significantly positively associated with HIF-1α and the HIF-1α-regulated gene VEGF. It is recognised that PHD2 and PHD3 expression is modulated by directly by hypoxia, but less appreciated is that PHD1 levels may be altered through suppression of its mRNA under hypoxia [29]. This is in keeping with the upregulation of all PHDs in the breast tumours in this series. Although one might anticipate that elevated levels of PHDs would lead directly to lower HIF through proteosomal degradation, the final effect is not predictable because, if chronic tumour hypoxia still persists, PHD hydroxylation will be abrogated. Furthermore, even in the presence of oxygen, there is some evidence to suggest that the scaffold protein map organiser 1 may block the hydroxylated HIF-1α from entering the degradation pathway, thus retaining some of its transcriptional activity [30]. Other mechanisms may also be present that may enable HIF to escape proteosomal degradation, such as generation of reactive oxygen species [31]. The absence of a correlation of PHDs with CAIX is likely to be due to its significantly longer half-life than HIF-1α, in the order of ~35 hours [32]. This is because both markers need to be present at the same time for an association to be identified. Thus, unlike HIF-1α that has a half-life measured in minutes, the prolonged half-life of CAIX means it would remain in the tumour when other proteins with a shorter half-life are no longer present.

We also observed a significant increase all PHDs post chemotherapy in both trial arms. This increase is potentially hypoxically mediated, since epirubicin has been reported to result in a reduction of blood flow and to have anti-angiogenic effects [33]. Furthermore, despite all PHDs generally increasing post chemotherapy, there was a general trend for upregulation of PHD2 and downregulation of PHD1 and PHD3 to be associated with response. Although this differential regulation is unexpected, one should note that although widely expressed they have differing tissue distributions (for example, PHD3 is highly expressed in the heart whereas PHD1 is highly expressed in the testes) and that it is unclear why the three different isoforms exist and what their specific or overlapping activities might be in HIF-related and HIF-independent biology.

Although all three PHDs may have similar roles in some biological functions, they thus also have function specific to individual PHDs. For example, PHD2 appears to be the sole PHD responsible for myocardial development since Phd2^-/- ^knockouts are embryonic lethal and the myocardium is severely underdeveloped, amongst other major defects [34]. The same phenotype is not observed with Phd1^-/- ^or Phd3^-/- ^knockouts. Furthermore, PHD3 is the only PHD that appears to mediate apoptosis [35], the latter requiring the catalytic activity of PHD3 (as cells are not rescued by HIF-1α and/or HIF-2α), suggesting that PHD3 has non-HIF targets [35]. The effects of PHDs, however, also appear to be cell type specific. The PHDs thus promote cell survival rather than cell death in chondrocytes. A further level of complexity is through the ability of particular PHDs to modulate HIF-independent pathways. PHD1, through hydroxylation of IκB kinase-β, thus fails to result in disassociation of IκB from NF-κB, altering this transcriptional response [36].

The above data support the notion that the resultant decrease in PHD1 could result in a tumour responding via HIF-independent mechanisms. Loss of PHD1 may thus be anticipated to downregulate cyclin D_1 _levels and suppress mammary tumour proliferation [37]. However, the biological effect of the expression of particular PHDs in individual tumours may also depend on the type of hypoxia (acute and/or chronic) to which it is exposed. Desensitisation and loss of HIF-1α (and HIF-2α) via hydroxylation by the PHDs has thus been reported to occur under long-term hypoxia, as intracellular oxygen availability is increased by inhibiting mitochondrial respiration [38].

## Conclusions

PHDs are frequently expressed in breast cancer, with PHD2 and PHD3 being the dominant isoforms. The association with HIF and VEGF and their upregulation on therapy in residual tumour suggests the PHDs may be a suitable target for anticancer therapy. Conflicting *in vivo *preclinical evidence for their differing functional effects in a variety of pathways, however, demonstrates that further preclinical work is needed to resolve these issues.

## Abbreviations

CAIX: carbonic anhydrase IX; ER: oestrogen receptor; HIF: hypoxia-inducible factor; NF: nuclear factor; PHD: prolyl hydroxylase; VEGF: vascular endothelial growth factor.

## Competing interests

The authors declare that they have no competing interests.

## Authors' contributions

SBF contributed to study conception and design, data analysis and interpretation, and writing the manuscript. DG contributed to study conception and design, collection and assembly of data, data analysis and interpretation, and writing the manuscript. AB contributed to study conception and design, collection and assembly of data, data analysis and interpretation, and writing the manuscript. MPB contributed to study conception and design, and collection and assembly of data. LC, SA, AB and LD contributed to the provision of study materials or patients. SB, GA, and TM contributed to the provision of study materials or patients, and collection and assembly of data. MM contributed to study conception and design, the provision of study materials or patients, and collection and assembly of data. ABo contributed to study conception and design. ALH contributed to data analysis and interpretation, and writing the manuscript. All authors read and approved the final manuscript.

## Supplementary Material

Additional file 1 **Supplementary data**. Table S1 presenting patient characteristics. Table S2 presenting the distribution of clinical and immunohistochemical parameters according to PHD1 expression (0, >0). Table S3 presenting the distribution of clinical and immunohistochemical parameters according to PHD2 expression (0, 1, ≥2). Table S4 presenting the distribution of clinical and immunohistochemical parameters according to PHD3 expression (0, 1, ≥2). Table S5 presenting the distribution of clinical and immunohistochemical parameters according to PHD (all positive). Figure S1 showing a consort diagram. Click here for file

## References

[B1] Harris AL Hypoxia - a key regulatory factor in tumour growth Nat Rev Cancer 2002 2 38 47 10.1038/nrc704 11902584

[B2] Generali D Berruti A Brizzi MP Campo L Bonardi S Wigfield S Bersiga A Allevi G Milani M Aguggini S Gandolfi V Dogliotti L Bottini A Harris AL Fox SB Hypoxia-inducible factor-1α expression predicts a poor response to primary chemoendocrine therapy and disease-free survival in primary human breast cancer Clin Cancer Res 2006 12 4562 4568 10.1158/1078-0432.CCR-05-2690 16899602

[B3] Maxwell PH The HIF pathway in cancer Semin Cell Dev Biol 2005 16 523 530 10.1016/j.semcdb.2005.03.001 16144689

[B4] Jaakkola P Mole DR Tian YM Wilson MI Gielbert J Gaskell SJ Kriegsheim Av Hebestreit HF Mukherji M Schofield CJ Maxwell PH Pugh CW Ratcliffe PJ Targeting of HIF-α to the von Hippel-Lindau ubiquitylation complex by O_2_-regulated prolyl hydroxylation Science 2001 292 468 472 10.1126/science.1059796 11292861

[B5] Maxwell PH Wiesener MS Chang GW Clifford SC Vaux EC Cockman ME Wykoff CC Pugh CW Maher ER Ratcliffe PJ The tumour suppressor protein VHL targets hypoxia-inducible factors for oxygen-dependent proteolysis Nature 1999 399 271 275 10.1038/20459 10353251

[B6] Epstein AC Gleadle JM McNeill LA Hewitson KS O'Rourke J Mole DR Mukherji M Metzen E Wilson MI Dhanda A Tian YM Masson N Hamilton DL Jaakkola P Barstead R Hodgkin J Maxwell PH Pugh CW Schofield CJ Ratcliffe PJ *C. elegans *EGL-9 and mammalian homologs define a family of dioxygenases that regulate HIF by prolyl hydroxylation Cell 2001 107 43 54 10.1016/S0092-8674(01)00507-4 11595184

[B7] Bos R van der Groep P Greijer AE Shvarts A Meijer S Pinedo HM Semenza GL van Diest PJ van der Wall E Levels of hypoxia-inducible factor-1α independently predict prognosis in patients with lymph node negative breast carcinoma Cancer 2003 97 1573 1581 10.1002/cncr.11246 12627523

[B8] Dales JP Garcia S Meunier-Carpentier S Andrac-Meyer L Haddad O Lavaut MN Allasia C Bonnier P Charpin C Overexpression of hypoxia-inducible factor HIF-1α predicts early relapse in breast cancer: retrospective study in a series of 745 patients Int J Cancer 2005 116 734 739 10.1002/ijc.20984 15849727

[B9] Chia SK Wykoff CC Watson PH Han C Leek RD Pastorek J Gatter KC Ratcliffe P Harris AL Prognostic significance of a novel hypoxia-regulated marker, carbonic anhydrase IX, in invasive breast carcinoma J Clin Oncol 2001 19 3660 3668 11504747 10.1200/JCO.2001.19.16.3660

[B10] Tan EY Campo L Han C Turley H Pezzella F Gatter KC Harris AL Fox SB BNIP3 as a progression marker in primary human breast cancer; opposing functions in in situ versus invasive cancer Clin Cancer Res 2007 13 467 474 10.1158/1078-0432.CCR-06-1466 17255267

[B11] Ryan HE Lo J Johnson RS HIF-1 α is required for solid tumor formation and embryonic vascularization EMBO J 1998 17 3005 3015 10.1093/emboj/17.11.3005 9606183 PMC1170640

[B12] Ryan HE Poloni M McNulty W Elson D Gassmann M Arbeit JM Johnson RS Hypoxia-inducible factor-1α is a positive factor in solid tumor growth Cancer Res 2000 60 4010 4015 10945599

[B13] Carmeliet P Dor Y Herbert JM Fukumura D Brusselmans K Dewerchin M Neeman M Bono F Abramovitch R Maxwell P Koch CJ Ratcliffe P Moons L Jain RK Collen D Keshert E Role of HIF-1α in hypoxia-mediated apoptosis, cell proliferation and tumour angiogenesis Nature 1998 394 485 490 10.1038/28867 9697772

[B14] Chan DA Kawahara TL Sutphin PD Chang HY Chi JT Giaccia AJ Tumor vasculature is regulated by PHD2-mediated angiogenesis and bone marrow-derived cell recruitment Cancer Cell 2009 15 527 538 10.1016/j.ccr.2009.04.010 19477431 PMC2846696

[B15] Soilleux EJ Turley H Tian YM Pugh CW Gatter KC Harris AL Use of novel monoclonal antibodies to determine the expression and distribution of the hypoxia regulatory factors PHD-1, PHD-2, PHD-3 and FIH in normal and neoplastic human tissues Histopathology 2005 47 602 610 10.1111/j.1365-2559.2005.02280.x 16324198

[B16] Bottini A Berruti A Brizzi MP Bersiga A Generali D Allevi G Aguggini S Bolsi G Bonardi S Tondelli B Vana F Tampellini M Alquati P Dogliotti L Cytotoxic and antiproliferative activity of the single agent epirubicin versus epirubicin plus tamoxifen as primary chemotherapy in human breast cancer: a single-institution phase III trial Endocr Relat Cancer 2005 12 383 392 10.1677/erc.1.00945 15947110

[B17] World Health Organization WHO Handbook for Reporting Results of Cancer Treatment 1978 WHO Offset Publication. Geneva, Switzerland: World Health Organisation

[B18] Generali D Buffa FM Berruti A Brizzi MP Campo L Bonardi S Bersiga A Allevi G Milani M Aguggini S Papotti M Dogliotti L Bottini A Harris AL Fox SB Phosphorylated ERα, HIF-1α, and MAPK signaling as predictors of primary endocrine treatment response and resistance in patients with breast cancer J Clin Oncol 2009 27 227 234 10.1200/JCO.2007.13.7083 19064988

[B19] Bottini A Berruti A Bersiga A Brizzi MP Brunelli A Gorzegno G DiMarco B Aguggini S Bolsi G Cirillo F Filippini L Betri E Bertoli G Alquati P Dogliotti L p53 but not bcl-2 immunostaining is predictive of poor clinical complete response to primary chemotherapy in breast cancer patients Clin Cancer Res 2000 6 2751 2758 10914720

[B20] Bottini A Berruti A Bersiga A Brizzi MP Bruzzi P Aguggini S Brunelli A Bolsi G Allevi G Generali D Betri E Bertoli G Alquati P Dogliotti L Relationship between tumour shrinkage and reduction in Ki67 expression after primary chemotherapy in human breast cancer Br J Cancer 2001 85 1106 1112 10.1054/bjoc.2001.2048 11710821 PMC2375158

[B21] McCarty KS Jr Miller LS Cox EB Konrath J McCarty KS Sr Estrogen receptor analyses. Correlation of biochemical and immunohistochemical methods using monoclonal antireceptor antibodies Arch Pathol Lab Med 1985 109 716 721 3893381

[B22] Boddy JL Fox SB Han C Campo L Turley H Kanga S Malone PR Harris AL The androgen receptor is significantly associated with vascular endothelial growth factor and hypoxia sensing via hypoxia-inducible factors HIF-1a, HIF-2a, and the prolyl hydroxylases in human prostate cancer Clin Cancer Res 2005 11 7658 7663 10.1158/1078-0432.CCR-05-0460 16278385

[B23] Couvelard A O'Toole D Turley H Leek R Sauvanet A Degott C Ruszniewski P Belghiti J Harris AL Gatter K Pezzella F Microvascular density and hypoxia-inducible factor pathway in pancreatic endocrine tumours: negative correlation of microvascular density and VEGF expression with tumour progression Br J Cancer 2005 92 94 101 10.1038/sj.bjc.6602245 15558070 PMC2361752

[B24] Ruan K Song G Ouyang G Role of hypoxia in the hallmarks of human cancer J Cell Biochem 2009 107 1053 1062 10.1002/jcb.22214 19479945

[B25] Semenza GL Regulation of cancer cell metabolism by hypoxia-inducible factor 1 Semin Cancer Biol 2009 19 12 16 10.1016/j.semcancer.2008.11.009 19114105

[B26] Henze AT Riedel J Diem T Wenner J Flamme I Pouyseggur J Plate KH Acker T Prolyl hydroxylases 2 and 3 act in gliomas as protective negative feedback regulators of hypoxia-inducible factors Cancer Res 2009 70 357 366 10.1158/0008-5472.CAN-09-1876 20028863

[B27] Minamishima YA Moslehi J Padera RF Bronson RT Liao R Kaelin WG Jr A feedback loop involving the Phd3 prolyl hydroxylase tunes the mammalian hypoxic response in vivo Mol Cell Biol 2009 29 5729 5741 10.1128/MCB.00331-09 19720742 PMC2772748

[B28] Appelhoff RJ Tian YM Raval RR Turley H Harris AL Pugh CW Ratcliffe PJ Gleadle JM Differential function of the prolyl hydroxylases PHD1, PHD2, and PHD3 in the regulation of hypoxia-inducible factor J Biol Chem 2004 279 38458 38465 10.1074/jbc.M406026200 15247232

[B29] Tian YM Mole DR Ratcliffe PJ Gleadle JM Characterization of different isoforms of the HIF prolyl hydroxylase PHD1 generated by alternative initiation Biochem J 2006 397 179 186 10.1042/BJ20051996 16509823 PMC1479752

[B30] Fong GH Takeda K Role and regulation of prolyl hydroxylase domain proteins Cell Death Diff 2008 15 635 641 10.1038/cdd.2008.10 18259202

[B31] Kaelin WG Jr Ratcliffe PJ Oxygen sensing by metazoans: the central role of the HIF hydroxylase pathway Mol Cell 2008 30 393 402 10.1016/j.molcel.2008.04.009 18498744

[B32] Sobhanifar S Aquino-Parsons C Stanbridge EJ Olive P Reduced expression of hypoxia-inducible factor-1α in perinecrotic regions of solid tumors Cancer Res 2005 65 7259 7266 10.1158/0008-5472.CAN-04-4480 16103077

[B33] Maragoudakis ME Peristeris P Missirlis E Aletras A Andriopoulou P Haralabopoulos G Inhibition of angiogenesis by anthracyclines and titanocene dichloride Ann N Y Acad Sci 1994 732 280 293 10.1111/j.1749-6632.1994.tb24743.x 7526759

[B34] Takeda K Ho VC Takeda H Duan LJ Nagy A Fong GH Placental but not heart defects are associated with elevated hypoxia-inducible factor alpha levels in mice lacking prolyl hydroxylase domain protein 2 Mol Cell Biol 2006 26 8336 8346 10.1128/MCB.00425-06 16966370 PMC1636770

[B35] Lee S Nakamura E Yang H Wei W Linggi MS Sajan MP Farese RV Freeman RS Carter BD Kaelin WG Jr Schlisio S Neuronal apoptosis linked to EglN3 prolyl hydroxylase and familial pheochromocytoma genes: developmental culling and cancer Cancer Cell 2005 8 155 167 10.1016/j.ccr.2005.06.015 16098468

[B36] Cummins EP Berra E Comerford KM Ginouves A Fitzgerald KT Seeballuck F Godson C Nielsen JE Moynagh P Pouyssegur J Taylor CT Prolyl hydroxylase-1 negatively regulates IκB kinase-β, giving insight into hypoxia-induced NFκB activity Proc Natl Acad Sci USA 2006 103 18154 18159 10.1073/pnas.0602235103 17114296 PMC1643842

[B37] Zhang Q Gu J Li L Liu J Luo B Cheung HW Boehm JS Ni M Geisen C Root DE Polyak K Brown M Richardson AL Hahn WC Kaelin WG Jr Bommi-Reddy A Control of cyclin D_1 _and breast tumorigenesis by the EglN2 prolyl hydroxylase Cancer Cell 2009 16 413 424 10.1016/j.ccr.2009.09.029 19878873 PMC2788761

[B38] Ginouves A Ilc K Macias N Pouyssegur J Berra E PHDs overactivation during chronic hypoxia 'desensitizes' HIFα and protects cells from necrosis Proc Natl Acad Sci USA 2008 105 4745 4750 10.1073/pnas.0705680105 18347341 PMC2290777

