# Supplementary material for: The prolyl hydroxylase enzymes are positively associated with hypoxia-inducible factor-1α and vascular endothelial growth factor in human breast cancer and alter in response to primary systemic treatment with epirubicin and tamoxifen
Source: Breast Cancer Res. 2011 Feb 3;13(1):R16. doi: 10.1186/bcr2825 (PMC3109585; doi:10.1186/bcr2825)
Supplement: Additional file 1 — Supplementary data. Table S1 presenting patient characteristics. Table S2 presenting the distribution of clinical and immunohistochemical parameters according to PHD1 expression (0, >0). Table S3 presenting the distribution of clinical and immunohistochemical parameters according to PHD2 expression (0, 1, ≥2). Table S4 presenting the distribution of clinical and immunohistochemical parameters according to PHD3 expression (0, 1, ≥2). Table S5 presenting the distribution of clinical and immunohistochemical parameters according to PHD (all positive). Figure S1 showing a consort diagram. [file bcr2825-S1.DOC]

Additional file 1

Table S1. Patient characteristics (n=187)

Randomisation

EPI 90 (48.1%)

EPI-TAM 97 (51.9%)

TNM

T2 144 (77.0%)

T3-4 43 (23.0%)

N0 106 (56.7%)

N1 81 (43.3%)

Tumour grade

2 49 (26.6%)

3 135 (73.4%)

Missing 3

Steroid hormone receptor status

ERα+ 147 (79.0%)

ERα- 39 (20.1%)

PgR+ 90 (48.4%)

PgR- 96 (51.6%)

Missing 1

Apoptosis markers

p53+ 93 (50.0%)

p53- 93 (50%)

bcl2- 51 (27.4%)

bcl2+ 135 (72.6%)

missing 1

**c-erbB2 status**

c-erbB2- 105 (56.1%)

c-erbB2+ 82 (43.9%)

Proliferative activity

Ki67 <10 48 (25.6%)

Ki67 10-20 106 (56.7%)

Ki67 >20 33 (17.6%)

Table S2. Distribution of clinical and immunohistochemical parameters according to PHD1 expression (0, >0)

|  |  | **0** |  | **>0** |  | **p value** |
| --- | --- | --- | --- | --- | --- | --- |
| **Grade** |  |  |  |  |  |  |
| 2 |  | 28/125 (22.4%) |  | 18/46 (39.1%) |  | <0.03 |
| 3 |  | 97/125 (97.6%) |  | 28/46 (60.9%) |  |  |
| **p53+** |  | 64/128 (50.0%) |  | 22/47 (46.8%) |  | 0.70 |
| **c-erbB2+** |  | 56/129 (43.4%) |  | 22/47 (46.8%) |  | 0.70 |
| **bcl2**+ |  | 97/128 (75.8%) |  | 34/47 (72.3%) |  | 0.64 |
| **ER**α**+** |  | 101/128 (78.9%) |  | 41/47 (87.2%) |  | 0.21 |
| **PgR**+ |  | 64/128 (50.0%) |  | 22/47 (46.8%) |  | 0.70 |
| **T2** |  | 97/129 (75.2%) |  | 38/47 (80.8%) |  | 0.43 |
| **T3-4** |  | 32/129 (24.8%) |  | 38/47 (80.8%) |  |  |
| **N+** |  | 57/129 (41.2%) |  | 20/47 (42.5%) |  | 0.84 |
| **Ki67** | **(mean)** | 23.4 |  | 18.5 |  |  |
|  | **(95%CI)** | 19.9 – 26.9 |  | 13.0-23.5 |  |  |

CI – confidence intervals

Table S3. Distribution of clinical and immunohistochemical parameters according to PHD2 expression (0, 1, ≥2).

|  |  | **0** |  | **1** |  | ≥**2** |  | **p value** |
| --- | --- | --- | --- | --- | --- | --- | --- | --- |
| **Grade** |  |  |  |  |  |  |  |  |
| 2 |  | 23/76 (30.3) |  | 14/51 (27.4) |  | 6/33 (18.2) |  | 0.21 |
| 3 |  | 53/76 (69.7) |  | 37/51 (72.5) |  | 27/33 (81.8) |  |  |
| **p53+** |  | 38/78 (48.7) |  | 22/50 (40.0) |  | 20/34 (58.8) |  | 0.45 |
| **c-erbB2+** |  | 33/78 (42.3) |  | 20/51 (39.2) |  | 19/34 (55.9) |  | 0.27 |
| **bcl2**+ |  | 60/78 (76.9) |  | 38/50 (76.0) |  | 21/34 (61.8) |  | 0.13 |
| **ER**α**+** |  | 63/78 (80.8) |  | 44/50 (88.0) |  | 22/34 (64.7) |  | 0.14 |
| **PgR**+ |  | 39/78 (50.0) |  | 25/50 (50.0) |  | 14/34 (41.2) |  | 0.45 |
| **T2** |  | 58/78 (74.4) |  | 40/51 (78.4) |  | 27/34 (79.4) |  | 0.51 |
| **T3-4** |  | 20/78 (25.6) |  | 11/51 (20.6) |  | 7/34 (20.6) |  |  |
| **N+** |  | 38/78 (48.7) |  | 19/51 (37.2) |  | 14/34 (41.2) |  | 0.19 |
| **Ki67** | **(mean)** | 21.7 |  | 20.6 |  | 25.6 |  | 0.48 |
|  | **(95%CI)** | 17.4-26.0 |  | 15.8-25.5 |  | 18.0-33.2 |  |  |

CI – confidence intervals

**Table S4.** Distribution of clinical and immunohistochemical parameters according to PHD3 expression (0, 1, ≥2).

|  |  | **0** |  | **1** |  | ≥**2** |  | **p value** |
| --- | --- | --- | --- | --- | --- | --- | --- | --- |
| **Grade** |  |  |  |  |  |  |  |  |
| 2 |  | 26/105 (24.8%) |  | 13/51 (25.5%) |  | 7/16 (43.7%) |  | 0.04 |
| 3 |  | 79/105 (75.2%) |  | 38/51 (74.5%) |  | 9/16 (56.3%) |  |  |
| **p53**+ |  | 59/108 (54.6%) |  | 21/52 (40.4%) |  | 7/16 (43.7%) |  | 0.13 |
| **c-erbB2** |  | 46/108 (42.6%) |  | 25/53 (47.2%) |  | 7/16 (43.7%) |  | 0.72 |
| **bcl2** |  | 85/108 (78.7%) |  | 33/53 (62.3%) |  | 13/15 (86.7%) |  | 0.45 |
| **ER**α**+** |  | 86/108 (79.6%) |  | 42/52 (80.8%) |  | 14/16 (87.5%) |  | 0.51 |
| **PgR** |  | 51/108 (47.2%) |  | 28/52 (53.8%) |  | 7/16 (43.7%) |  | 0.82 |
| **T2** |  | 80/108 (74.1%) |  | 42/53 (79.3%) |  | 14/16 (87.5%) |  | 0.2 |
|  |  | 28/108 (25.9%) |  | 11/53 (20.7%) |  | 2/16 (12.5%) |  |  |
| **N+** |  | 46/108 (42.6%) |  | 22/53 (41.5%) |  | 10/16 (62.5%) |  | 0.29 |
| **Ki67** | **(mean)** | 23 |  | 21.7 |  | 17.4 |  | 0.47 |
|  | **(95%CI)** | (19.3-26.7) |  | (16.0-26.4) |  | (12.5-22.2) |  |  |

CI – confidence intervals

**Table S5.** Distribution of clinical and immunohistochemical parameters according to PHD (all positive).

|  |  | **Negative** |  | **Positive** |  | **p value** |
| --- | --- | --- | --- | --- | --- | --- |
| **Grade** |  |  |  |  |  |  |
| 2 |  | 23/76 (30.3) |  | 14/51 (27.4) |  | 0.21 |
| 3 |  | 53/76 (69.7) |  | 37/51 (72.5) |  |  |
| **p53+** |  | 38/78 (48.7) |  | 22/50 (40.0) |  | 0.45 |
| **c-erbB2+** |  | 33/78 (42.3) |  | 20/51 (39.2) |  | 0.27 |
| **bcl2**+ |  | 60/78 (76.9) |  | 38/50 (76.0) |  | 0.13 |
| **ER**α**+** |  | 63/78 (80.8) |  | 44/50 (88.0) |  | 0.14 |
| **PgR**+ |  | 39/78 (50.0) |  | 25/50 (50.0) |  | 0.45 |
| **T2** |  | 122/137 (89.0) |  | 38/41 (92.7) |  | 0.48 |
| **T3-4** |  | 15/137 (10.9) |  | 3/41 (7.3) |  |  |
|  |  |  |  |  |  |  |
|  |  |  |  |  |  |  |
| **N+** |  | 38/78 (48.7) |  | 19/51 (37.2) |  | 0.19 |
| **Ki67** | **(mean)** | 21.7 |  | 20.6 |  | 0.48 |
|  | **(95%CI)** | 17.4-26.0 |  | 15.8-25.5 |  |  |

CI – confidence intervals

**Figure 1: Consort Diagram**


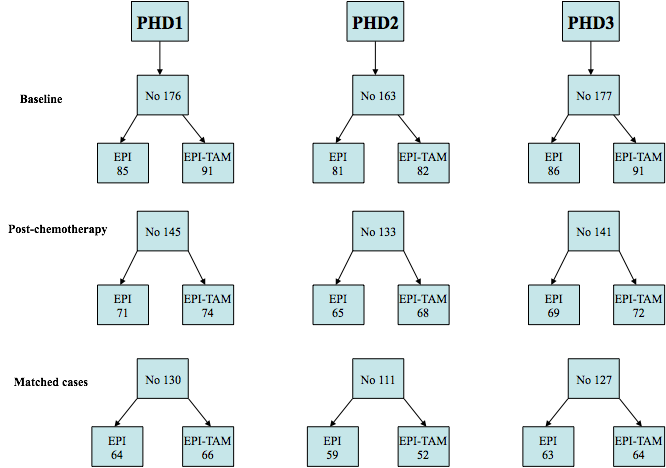


For HIF-1 alpha, 9 of the cores were absent and 7 were without sufficient number of tumor cells to score. For CAIX, 21 cases were missing.15 out of 21 the cores were absent and 6 were without sufficient number of tumor cells to score. For VEGF, 27 cases were missing. 12 out of 27 the were absent and 15 were without sufficient number of tumor cells to score. For PHD1, 11 cases were missing. 3 out of 11 the cores were absent and 8 were without sufficient number of tumor cells to score. For PHD2, 24 cases were missing. 12 out of 24 the cores were absent and 12 were without sufficient number of tumor cells to score. For PHD3, 10 cases were missing. 3 out of 10 the cores were absent and 7 were without sufficient number of tumor cells to score.
